# Expression of IkappaB Family in the Ovine Liver during Early Pregnancy

**DOI:** 10.3390/ani13061057

**Published:** 2023-03-15

**Authors:** Chunjiang Cai, Ying Ren, Jianhua Cao, Shengya Fang, Leying Zhang, Ling Yang

**Affiliations:** School of Life Sciences and Food Engineering, Hebei University of Engineering, Handan 056038, China

**Keywords:** IkappaB, liver, pregnancy, sheep

## Abstract

**Simple Summary:**

An inhibitor of the NF-κB (IκB) family is involved in regulating innate immune responses. In this study, early pregnancy induces upregulation of IκBα, but changes and downregulates the expression of BCL-3, IκBε, IKKγ and IκBNS, and changes expression of IκBβ and IκBζ in the maternal liver, which are related to maternal peripheral tolerance and pregnancy establishment.

**Abstract:**

During normal pregnancy, there is a dynamic regulation of the maternal immune system, including the liver, to accommodate the presence of the allogeneic foetus in the uterus. However, it was unclear that the expression of the IkappaB (IκB) family was regulated in the ovine maternal liver during early pregnancy. In this study, sheep livers were collected at day 16 of the oestrous cycle (NP16), and days 13, 16 and 25 of gestation (DP13, DP16 and DP25), and RT-qPCR, Western blot and immunohistochemistry analysis were used to analyse the expression of the IκB family, including B cell leukemia-3 (BCL-3), IκBα, IκBβ, IκBε, IKKγ, IκBNS and IκBζ. The results revealed that expression of BCL-3, IκBβ, IκBε and IKKγ peaked at DP16, and the expression of IκBα was increased during early pregnancy. In addition, the expression of IκBζ peaked at DP13 and DP16, and IκBNS peaked at DP13. IκBβ and IKKγ proteins were located in the endothelial cells of the proper hepatic arteries and portal veins, and hepatocytes. In conclusion, early pregnancy changed the expression of the IκB family, suggesting that the modulation of the IκB family may be related to the regulation of maternal hepatic functions, which may be favourable for pregnancy establishment in sheep.

## 1. Introduction

During normal pregnancy in humans, there are dynamic changes in the peripheral maternal immune system, which are necessary for the maternal immune system to maintain tolerance towards the allogeneic foetus [1]. The maternal immune system undergoes major adaptive modifications that result in multiple immunological-associated changes in immune cell populations with the time of pregnancy in humans [2]. During early pregnancy in sheep, the embryo regulates the gene expression of the maternal immune system with pregnancy status, which is essential for the successful establishment of the pregnancy [3]. Conceptus signalling (interferon-tau, IFNT) works in parallel with the pattern recognition receptors to modulate the maternal innate immune system and prevent conceptus rejection through paracrine and endocrine manners during early pregnancy in ruminants [4]. There is modulation of interferon-stimulated genes (ISGs) and progesterone receptors in maternal immune organs, including bone marrow [5,6], the thymus [7,8], the spleen [9,10,11] and lymph nodes [12,13,14], which are regulated by IFNT and progesterone through an endocrine manner during early pregnancy in sheep.

As an immunological organ, the liver promotes self-tolerance through inhibition of peripheral T cells, and the optimal protective adaptive immune responses are related with the modulation of hepatic innate immune cells in the host [15]. Reproductive state and pregnancy hormones, including thyroid hormone, oestrogen and progesterone, modulate liver size and function during pregnancy in humans and rodents [16]. There are pregnancy-related hypertrophy and hyperproliferation of hepatocytes, which induce increases in stiffness and water diffusion, and decreases in viscosity in ex vivo rat liver specimens obtained from rats with normal pregnancy [17]. During pregnancy in rats, hepatic insulin-like growth factor I (IGF-I) mRNA level is low, and IGF binding protein 4 (IGFBP-4) mRNA level is downregulated at days 14 and 21 of pregnancy [18]. It has been reported that in the ovine maternal liver, early pregnancy regulates the expression of gonadotropin releasing hormone and prolactin, and their receptors, prostaglandin synthases, T helper (Th) cytokines, melatonin receptor 1, CD4, [19,20,21,22,23]. Furthermore, toll-like receptor pathway, nuclear factor kappa B (NF-κB) pathway and complement pathway are modulated in the ovine maternal liver during early pregnancy [24,25,26].

NF-κB regulates over 400 genes related to inflammation, apoptosis and angiogenesis that are associated with maternal immunosuppression and maintaining gestation. Furthermore, the expression of NF-κB components, including NF-κB p105, NF-κB p100, p65, RelB and c-Rel, is changed in the maternal immune organs in ewes, which are related to the maternal immunoregulation, embryo implantation and pregnancy maintenance in sheep [26,27,28,29]. Inhibitors of the NF-κB (IκB) family include IκBα, IκBβ, IκBε, IKKγ, B cell leukemia-3 (BCL-3), IκBNS (also known as NFKBID) and IκBζ, which participate in the regulation of innate immune responses [30]. As negative nuclear regulators of NF-κB, IκBNS and BCL-3 modulate transcription of interleukin-6 (IL-6) and tumour necrosis factor (TNF)-α which are essential cytokines for blastocyst implantation in mice [31]. The activation of IκBα and IκBβ in peripheral blood mononuclear cells is regulated in pregnancy, which plays a key role in maternal immune regulation, and the foetus avoiding maternal rejection [32]. IKKβ is implicated in oxytocin-induced NF-κB-p65 phosphorylation in the myometrium and amnion in human term/preterm labour [33]. Therefore, IκB proteins are involved in pregnancy maintenance. It was hypothesized that the expression of IκB proteins was regulated in the ovine liver during early pregnancy. The aim of this research was to analyse the gene and protein expression of the IκB family in the ovine maternal liver during early pregnancy, which will be helpful for making out the modulation of maternal hepatic function during early pregnancy in sheep.

## 2. Materials and Methods

### 2.1. Animal Tissue Collection

Healthy Small-tail Han ewes (approximately 18-month-old, similar genetic background, average weight of 41 kg and normal oestrus) were chosen and housed using conventional breeding and nutrition, and free access to food and water in a local ovine farm (Handan, China). The ewes were oestrus-synchronised using a progesterone-releasing intravaginal device, and divided into four groups (n = 6 for each group) at random. A teaser ram was used to detect oestrus according to obvious oestrous signs (day 0), and the ewes of three groups were bred with intact rams, and the nonpregnant ewes (other group) were not mated with an intact ram. Livers were collected on days 13, 16 and 25 for pregnant animals (DP13, DP16 and DP25), and day 16 of the oestrous cycle (NP16) for nonpregnant females after the ewes were killed. Pregnancy was validated by anatomical observation of an embryo in the uterus. Liver tissues were immediately immersed in fresh 4% (*w*/*v*) paraformaldehyde, or stored at −80 °C for real-time quantitative PCR (RT-qPCR) and protein expression analysis.

### 2.2. RT-qPCR Assay

Hepatic tissue RNA extraction, concentration measurement, and cDNA synthesis were conducted as described previously [21]. RT-qPCR was performed using specific primers of BCL-3, NFKBIA, NFKBIB, NFKBIE, IKBKG, NFKBID and NFKBIZ on a Bio-Rad CFX96 real-time PCR system (Bio-Rad Laboratories, Hercules, CA, USA). The primers for the target genes and glyceraldehyde-3-phosphate dehydrogenase (GAPDH) (Table 1) were designed and synthesized by Shanghai Sangon Biotech Co., Ltd. (Shanghai, China), and GAPDH was amplified in parallel with the target genes. The PCR conditions were 40 cycles of 95 °C for 10 s, 60–62.5 °C (60 °C for BCL-3, 60.5 °C for NFKBIA, NFKBID and NFKBIZ, 61 °C for NFKBIB and NFKBIE, 62.5 °C for IKBKG) for 20 s, and 72 °C for 25 s. Three biological replicates were completed for all samples, and expression levels of the target genes were analysed using the 2^−ΔΔCt^ analysis method [34]. Relative expression levels were calculated using the cycle threshold from the ewes on day 16 of the oestrous cycle.

### 2.3. Western Blot

Protein isolation, concentration quantification and separation, as well as protein were transferred onto PVDF membranes (Millipore, Bedford, MA, USA), blocked with 5% fat-free milk, and membrane incubation was performed as described previously [21]. The primary antibodies included an anti-BCL-3 antibody (Santa Cruz Biotechnology, Santa Cruz, CA, USA, sc-32741), an anti-IκBα antibody (Santa Cruz Biotechnology, sc-1643), an anti-IκBβ antibody (Santa Cruz Biotechnology, sc-390622), an anti-IκBεantibody (Santa Cruz Biotechnology, sc-7275), an anti-IKKγ antibody (Santa Cruz Biotechnology, sc-166398), an anti-NFKBID antibody (Abcam, Cambridge, UK, ab232913), and an anti-IκBζ antibody (Abcam, ab155142). The antibodies were validated by ovine proteins, and suitable for sheep. Secondary antibody goat anti-mouse IgG-horseradish peroxidase-conjugated (HRP) (Biosharp, Tallinn, Estonia, BL001A) or goat anti-rabbit IgG-HRP (Biosharp, BL003A) was incubated in a 1:10 000 dilution. GAPDH (anti-GAPDH antibody, Santa Cruz Biotechnology, sc-20357, 1:1000) was used as the loading control. An enhanced chemiluminescence kit (Tiangen Biotech Co., Ltd., Beijing, China) was used to detect target proteins. The band intensity was quantified with Quantity One V452 software (Bio-Rad Laboratories, Hercules, CA, USA).

### 2.4. Immunohistochemistry Analysis

The fixed hepatic tissues were treated as described previously [26]. The anti-IκBβantibody (Santa Cruz Biotechnology, sc-390622, 1:200) and anti-IKKγantibody (Santa Cruz Biotechnology, sc-166398, 1:200) were used for immunohistochemical localization of IκBβ and IKKγ in the liver tissue. After being rinsed three times for 5 min, sections were incubated with goat anti-mouse IgG-HRP (Biosharp, Hefei, China, BL001A) in a 1:1000 dilution. The negative control was treated with goat anti-mouse IgG instead of IκBβ and IKKγ antibodies. A DAB kit (Tiangen Biotech Co., Ltd., Beijing, China) was used to visualize the antibody binding sites, and then the nucleus was stained with haematoxylin. Tissue sections were photographed using a light microscope (Nikon Eclipse E800, Tokyo, Japan) with a digital camera (AxioCam ERc 5s), and the intensity of staining was analysed through the photos by two investigators in a blinded fashion. The intensity of staining was scored on a scale of 0 to 3:0, no staining; 1, weak staining; 2, strong staining; 3, stronger staining, as described previously [24].

### 2.5. Statistical Analysis

MIXED procedure in SAS (Version 9.1; SAS Institute, Cary, NC, USA) was used for all statistical analyses. Data of the IκB family mRNA and proteins were from a population with a normal distribution, and analysed using the Duncan method for comparing the relative expression levels of the different groups. Data are presented as mean ± SEM. *p* < 0.05 was considered statistically significant.

## 3. Results

### 3.1. Gene Expression of IκB Family in the Liver

**Figure 1:**
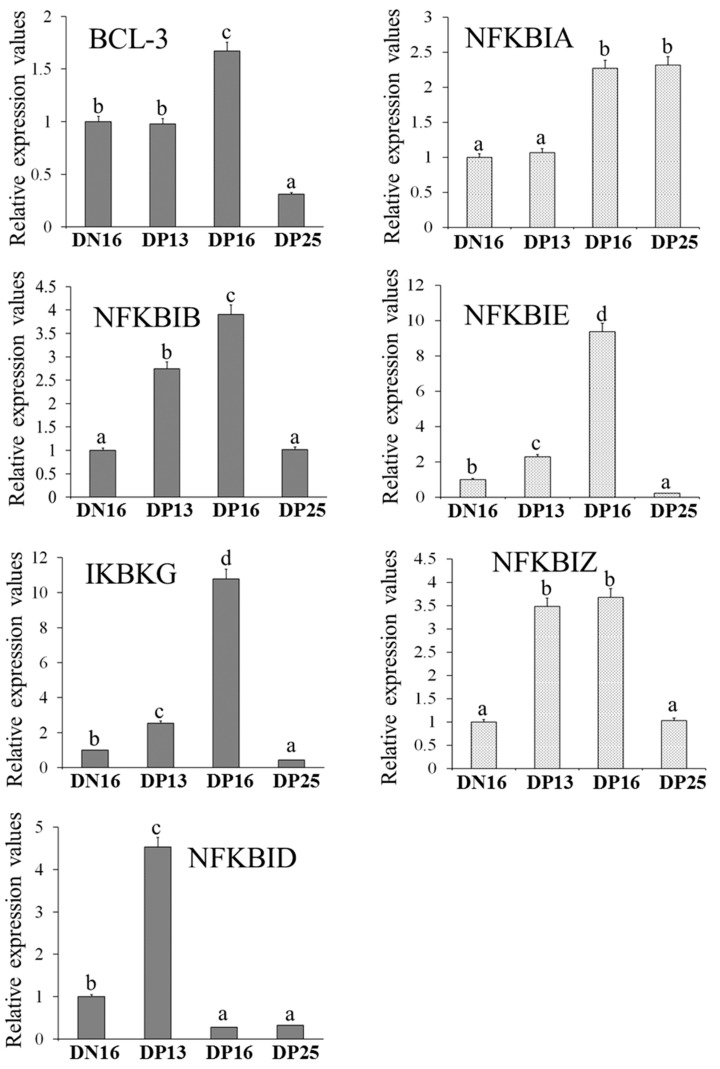
Relative expression values of BCL-3, NFKBIA, NFKBIB, NFKBIE, IKBKG, NFKBID and NFKBIZ mRNA in ovine liver. Note: DN16 = day 16 of the oestrous cycle; DP13 = day 13 of pregnancy; DP16 = day 16 of pregnancy; DP25 = day 25 of pregnancy. Significant differences (*p* < 0.05) are indicated by different letters within same colour column.

Figure 1 revealed that compared to NP16 and DP13, the relative expression level of BCL-3 mRNA was increased at DP16, but decreased significantly at DP25 (*p* < 0.05). The relative expression level of NFKBIA mRNA was upregulated at DP16 and DP25 compared toNP16 and DP13 (*p* < 0.05). Furthermore, there was an increase in the relative expression levels of NFKBIE and IKBKG mRNA from DP13 and DP16 compared to NP16, but the levels were downregulated significantly at DP25 (*p* < 0.05). In addition, NFKBIB and NFKBIZ mRNA levels were higher at DP13 and 16 compared to NP16 and DP25 (*p* < 0.05). On the other hand, there was a peak in the relative expression level of NFKBID mRNA at DP13, but the level of NFKBID was lower at DP16 and DP25 compared to NP16 (*p* < 0.05).

### 3.2. Protein Expression of IκB Family in the Livers

**Figure 2:**
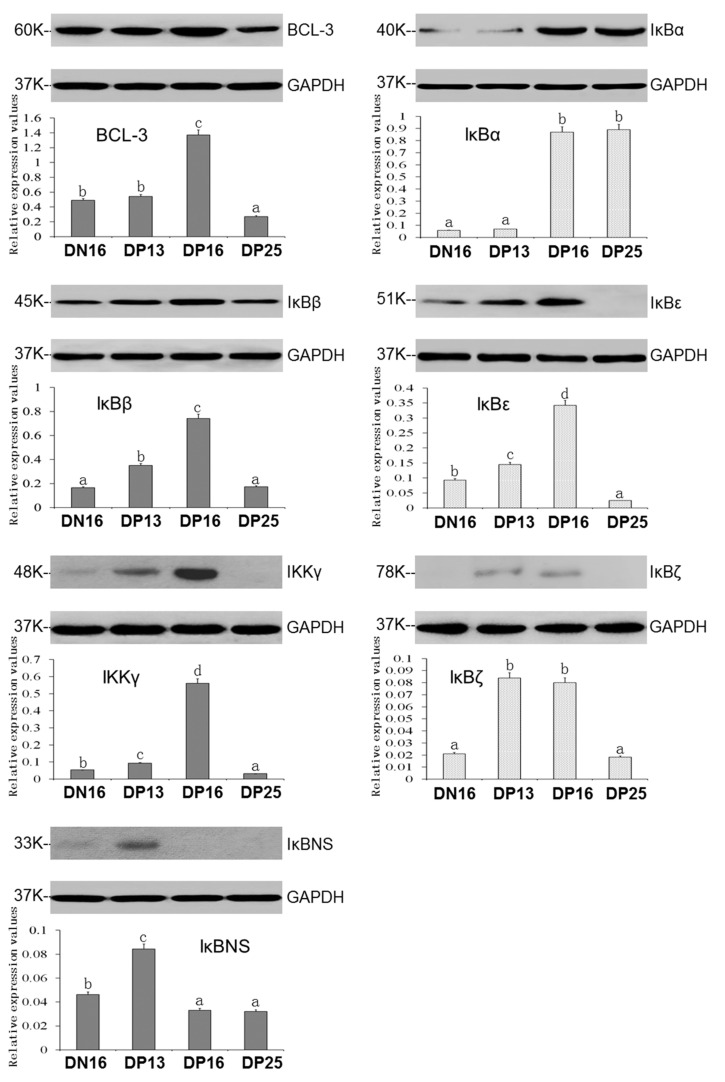
Expression of IκB proteins in ovine liver. Note: DN16 = day 16 of the oestrous cycle; DP13 = day 13 of pregnancy; DP16 = day 16 of pregnancy; DP25 = day 25 of pregnancy. Significant differences (*p* < 0.05) are indicated by different superscript letters within the same colour column.

It was shown in Figure 2 that BCL-3, IκBβ, IκBε and IKKγ protein levels were peaked at DP16, but BCL-3, IκBε and IKKγ levels were the lowest at DP25 (Figure 2; *p* < 0.05), and they were almost undetectable for IκBε and IKKγ proteins at DP25. The level of IκBα protein was higher at DP16 and DP25 than NP16 and DP13 (*p* < 0.05). In addition, the expression level of IκBζ protein was higher at DP13 and DP16, but IκBζ protein was almost undetected at NP16 and DP25 (*p* < 0.05). On the other hand, the expression level of IκBNS protein was the highest at DP13 (*p* < 0.05), but IκBNS protein was almost undetected at DP16 and DP25 (Figure 2).

### 3.3. Immunohistochemistry for IκBβ and IKKγ Proteins in the Livers

**Figure 3:**
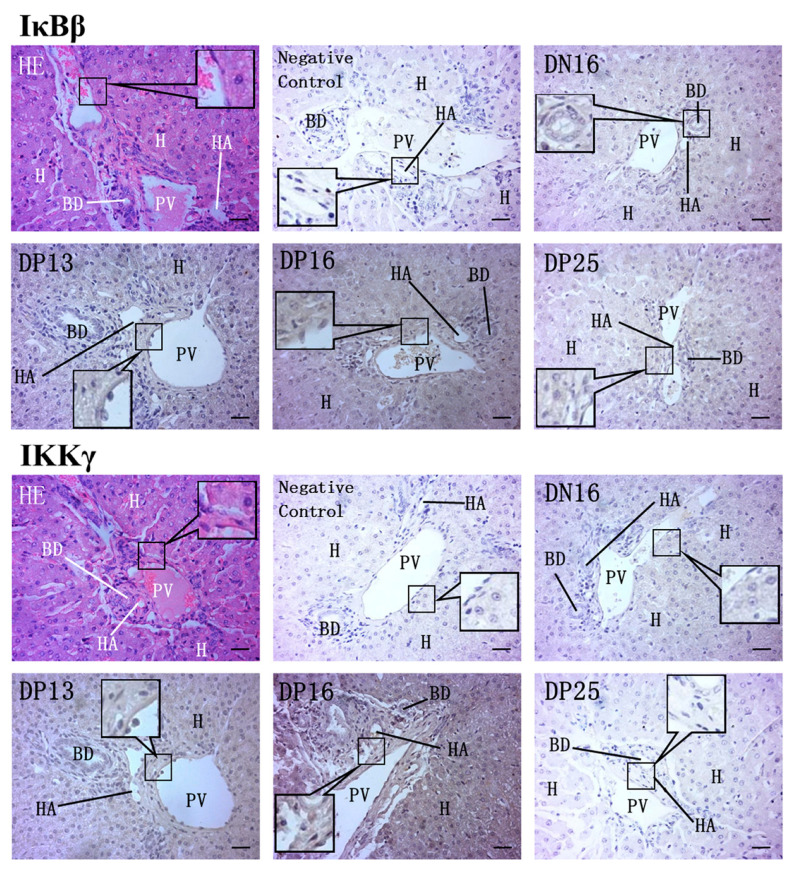
Representative immunohistochemical localization of IκBβ and IKKγ proteins in ovine liver. Liver is divided into lobes, and lobe is made up of hepatic lobules. A portal triad is a component of the hepatic lobule, consists of proper hepatic artery (HA), hepatic portal vein (PV), small bile ductile (BD). Note: HE = stained by haematoxylin and eosin; H = hepatocyte; DN16 = day 16 of nonpregnancy; DP13 = day 13 of pregnancy; DP16 = day 16 of pregnancy; DP25 = day 25 of pregnancy. Bar = 50 µm.

In the liver, IκBβ and IKKγ proteins were located in the endothelial cells of the proper hepatic arteries and portal veins, and hepatocytes. For the negative control, the livers from NP16, and livers from DP13, DP16 and DP25, and the staining intensities for IκBβ protein were negative, weak, strong, stronger and weak, and the staining intensities for IKKγ protein were negative, weak, strong, stronger and negative, respectively (Figure 3).

## 4. Discussion

BCL-3 either enhances or suppresses NF-κB target gene expression dependent on the type of cell, the type, and the type of NF-κB target genes implicated [35]. BCL-3 modulates the expression of specific genes to participate in suppression of the innate immune response, but overexpression of BCL-3 in human placentas is related to severe early-onset preeclampsia [36]. BCL-3 is implicated in the remodelling of the uterus for blastocyst implantation through negatively regulating TNF-α transcription, which is necessary for the dynamic regulation of NF-κB activity in the uterus to maintain a favourable environment of cytokines for pregnancy preparation [31]. BCL-3 is expressed in 7–10-day mouse embryo implantation sites, and detected over decidua at 7–8 days post coitum, but has weak labelling at 10 days post coitum [37]. In this study, early pregnancy induced upregulation of BCL-3 at DP16, but downregulation at DP25 in the maternal liver. Therefore, the changes in expression of BCL-3 may be related to the dynamic regulation of NF-κB activity in the maternal liver to maintain a favourable hepatic function for preparing pregnancy in sheep.

IκBα is an isoform of NF-κB inhibitor protein, and is involved in strong negative feedback to allow for a fast turn-off of the NF-κB response in gene expression [38]. Progesterone/progesterone receptor inhibits NF-κB activation and inflammation in the myometrium, which is via increasing the expression of cytoplasmic IκBα [39]. Progesterone receptors are upregulated in the maternal liver from pregnant ewes, which is implicated in the modulation of maternal hepatic immunoregulatory and other functions during early pregnancy in sheep [40]. Maternal vitamin D deficiency during pregnancy leads to IκBα methylation and a decrease in liver IκBα expression, as well as insulin resistance and declined inflammation in rat male offspring [41]. The activation of NF-κB in foeto-maternal uterine tissues is associated with preterm birth (PTB) pathophysiology, but IκBα treatment can reduce the inflammatory response related to PTB [42]. Our results revealed that early pregnancy induced expression of IκBα in the maternal liver. Therefore, the upregulation of IκBα in the maternal liver may be favourable for pregnancy establishment during early pregnancy in sheep.

An inhibitor of NF-κB family member IκBβ can attenuate the expression of select pro-inflammatory target genes, which results in weakening the pro-inflammatory response and exacerbating disease [43]. Although IκBβ is structurally similar to IκBα, the molecular interactivity of IκBβ with the kinase-active region of IKK subunit 2 and phosphorylation status differs markedly from IκBα [44]. The preeclamptic placentas have significantly higher IκBβ protein level, suggesting that NF-κB activation pathways are downregulated in preeclamptic placentas [45]. It was shown in this research that IκBβ level was increased on DP13 and DP16, but declined on DP25 in the maternal liver. In addition, IκBβ protein was located in the endothelial cells of the proper hepatic arteries and portal veins, and hepatocytes. Expression levels of ISG15 and signal transducer and activator of transcription 1 (STAT1) are significantly higher on days 13 and 16 of pregnancy, which is related to the IFNT (conceptus signalling) reaching the liver through blood or/and immune cells to affect the maternal hepatic immune response and other functions in ewes [46]. Early pregnancy induces the expression of NF-κB2, RelA and RelB proteins in the maternal liver with the pregnancy progress, which are associated with maintaining maternal liver homeostasis and immune tolerance in ewes [26]. Therefore, the upregulation of IκBβ on DP13 and DP16 may be related to the IFNT, but the downregulation of IκBβ on DP25 may be related to the upregulation of NF-κB2, RelA and RelB proteins in the maternal liver in sheep.

The IκB family suppresses DNA binding and localizing NF-κB factors to the cell cytoplasm, but unlike IκBα and IκBε they sequester RelA or c-Rel in the cytoplasm via inhibiting nuclear import [47]. IκBε regulates immune-response functions of B cells via at least two mechanisms involving cRel- and RelA-containing NF-κB dimers, but c-Rel induces only the IκBε gene [48]. The NF-κB members form homo- and heterodimers that respond to extracellular stress responses by turning on hundreds of genes, and dimers containing c-Rel prefer IκBε [49]. Early pregnancy suppresses c-Rel expression in the maternal liver, but c-Rel expression level is increased from day 13 to 25 of pregnancy in ewes [26]. In this study, we determined that IκBε was upregulated in the maternal liver at days 13 to 16 of pregnancy, but significantly downregulated at day 25 of pregnancy, and the expression pattern of IκBε was contrary to c-Rel expression in the maternal liver. The c-Rel plays a key role in the controlling liver homeostasis, and is necessary for hepatic inflammation, wound-healing, and hepatocyte proliferation in mice [50]. Therefore, upregulation of IκBε at DP13 and DP16 may be related to NF-κB suppression, but the downregulation of IκBε at DP25 may be favourable for liver homeostasis and regeneration through regulating c-Rel expression.

The IKK complex is a trimeric complex, including two kinases and a regulatory subunit Nemo (IKKγ) that is involved in regulating NF-κB pathways through phosphorylation and degradation of the NF-κB family [51]. Expression level of IKKγ gene is higher in the blood of women with preeclampsia compared to healthy controls, suggesting that an increase in NEMO gene expression in the mother is involved in the preeclampsia development [52]. IKKγ protein is mainly localized in the syncytiotrophoblast layer of placentas, and IKKγ increases the inflammatory state characteristic for preeclampsia and the necrosis within preeclamptic placentas [53]. IKKγ participates in hepatic NF-κB activation, and is a crucial regulator of the hepatic inflammatory response, hepatocyte survival and energy metabolism [54]. Our data revealed that IKKγ peaked at DP16, but significantly declined at DP25 comparing to nonpregnancy. On the other hand, IKKγ protein was located in the endothelial cells of the proper hepatic arteries and portal veins, and hepatocytes. Therefore, it is suggested that the peak of IKKγ mRNA and protein may be associated with the regulation of the hepatic inflammatory response, hepatocyte survival and energy metabolism, but significant downregulation of IKKγ at DP25 may be beneficial for pregnancy establishment in ewes.

IκB-ζ is the principal mediator downstream of NF-κB, and is involved in inflammation, oxidative stress and senescence [55]. IκBζ-dependent genes include IL-6 and lipocalin-2 that contribute to controlling acute hepatitis and bacterial infection [56]. Hepatic IκBζ regulates the factors related to triglyceride metabolism, which attenuates the progression of non-alcoholic fatty liver disease in mice [57]. Galectin-1 can downregulate IκBζ in the primary cultures of decidua cells, which is related with downregulation of the inflammatory response and the abundance of anti-inflammatory molecules during gestation [58]. There is an increase in expression levels of ISG15 and STAT1 in the maternal liver on days 13 and 16 of pregnancy, which is related to the IFNT from the conceptus in ewes [46]. Our data showed that IκBζ was upregulated in the maternal liver at DP13 and DP16, but downregulated at DP25. Therefore, the upregulation of IκBζ may be associated with inducible expression of hepatic ISGs by IFNT, but the downregulation at DP25 may contribute to the regulation of the maternal hepatic inflammatory response.

IκBNS improves follicular helper T-cell differentiation, which is implicated in the production of antigen-specific IgG through IL-21 induction [59]. The absence of IκBNS results in impaired plasma cell differentiation, an increase in mitochondrial metabolism and decrease in autophagic capacity [60]. IκBNS is an atypical NF-κB inhibitor, and also a potential target in regulating CD4+ T-cell activation, proliferation, and Th1-cell differentiation [61]. Our previous study showed that the expression of NF-κB components, including NF-κB1, NF-κB2, RelA, RelB and c-Rel, is lower at DP13 [26]. IκBNS negatively regulates transcription of IL-6 which plays key roles in the remodelling of the uterus for blastocyst implantation and the onset of labour during pregnancy under transcriptional control of NF-κB in mice [31,62]. Our data showed that IκBNS protein was strongly expressed at DP13 in the maternal liver, but then undetected at DP16 and DP25. Therefore, the upregulation of IκBNS at DP13 may be related to the peripheral tolerance, but the downregulation at days DP16 and DP25 may be associated with the blastocyst implantation during early pregnancy in sheep.

**Figure 4:**
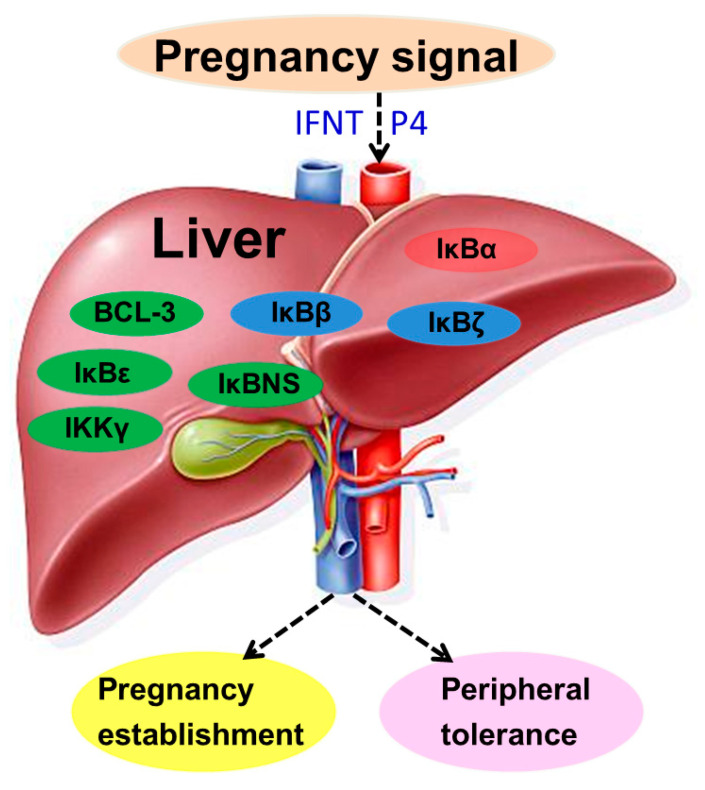
Sketch of IκB family in the liver during early pregnancy. Early pregnancy signals (interferon-tau (IFNT) and progesterone (P4)) change expression of IkappaB (IκB) family, which leads to maternal peripheral tolerance and pregnancy establishment. IκB family includes B cell leukemia-3 (BCL-3), IκBα, IκBβ, IκBε, IKKγ, IκBNS and IκBζ. Red, increase; Green, changed and decrease; Blue, changed.

During early pregnancy in ewes, early pregnancy signals (IFNT and progesterone) enhance the expression of IκBα but modulate and suppress the expression of BCL-3, IκBε, IKKγ and IκBNS, and regulate the expression of IκBβ and IκBζ in the maternal liver through blood circulation, which are associated with maternal peripheral tolerance and pregnancy establishment (Figure 4).

## 5. Conclusions

Early pregnancy changed the expression of IκB family, and modulated the expression of IκBβ and IKKγ proteins in the endothelial cells of the proper hepatic arteries and portal veins, and hepatocytes, which may be related to the dynamic regulation of NF-κB activity in the maternal liver. There were increases in the expression of the IκB family at DP13 and/or DP16, which may be associated with the IFNT from conceptus and progesterone from the corpus lutea. These changes in expression of the IκB family may be involved in the regulation of maternal hepatic homeostasis and regeneration, the inflammatory response and peripheral tolerance, which may be beneficial for pregnancy establishment in sheep.

## Figures and Tables

**Table 1:** Primers used for RT-qPCR.

Gene	Primer	Sequence	Size (bp)	Accession Numbers
BCL-3	Forward	GCGACCAGAGGCAATTTACTACCAG	98	XM_027978453.2
	Reverse	GAGGTGTAGGCAAGTTCAGCAGAG		
NFKBIA	Forward	AGGACGAGGAGTATGAGCAGATGG	130	NM_001166184.1
	Reverse	GCCAAGTGCAGGAACGAGTCTC		
NFKBIB	Forward	CCCCAAGACCTACCTCGCTCAG	119	XM_027978262.2
	Reverse	TCCAGTCCTCTTCACTCTCATCCTC		
NFKBIE	Forward	GCACTCACGTACATTTCCGAGGAC	97	XM_042236979.1
	Reverse	GCAGCAGAGCCAGGCAATACAG		
IKBKG	Forward	GGGCAACCAGAGGGAGGAGAAG	146	XM_027963334.2
	Reverse	GGCATGTCTTCAGGCGTTCCAC		
NFKBIZ	Forward	GCAAAGGCGTACAATGGAAACACC	137	NM_001306117.1
	Reverse	GGCTGCTCGTTCTCCAAGTTCC		
NFKBID	Forward	ACATTCGTGAGCATAAGGGCAAGAC	114	XM_027977435.2
	Reverse	GATGGTCAGTGGCATTGGGTTCC		
GAPDH	Forward	GGGTCATCATCTCTGCACCT	176	NM_001190390.1
	Reverse	GGTCATAAGTCCCTCCACGA		

## Data Availability

Not applicable.
